# An E–Mental Health Solution to Prevent and Manage Posttraumatic Stress Injuries Among First Responders in Alberta: Protocol for the Implementation and Evaluation of Text Messaging Services (Text4PTSI and Text4Wellbeing)

**DOI:** 10.2196/30680

**Published:** 2022-04-25

**Authors:** Gloria Obuobi-Donkor, Ejemai Eboreime, Jennifer Bond, Natalie Phung, Scarlett Eyben, Jake Hayward, Yanbo Zhang, Frank MacMaster, Steven Clelland, Russell Greiner, Chelsea Jones, Bo Cao, Suzette Brémault-Phillips, Kristopher Wells, Xin-Min Li, Carla Hilario, Andrew J Greenshaw, Vincent Israel Opoku Agyapong

**Affiliations:** 1 Department of Psychiatry Faculty of Medicine and Dentistry University of Alberta Edmonton, AB Canada; 2 Operational Stress Injury Clinic Alberta Health Services Edmonton, AB Canada; 3 Department of Emergency Medicine Faculty of Medicine and Dentistry University of Alberta Edmonton, AB Canada; 4 Department of Psychiatry Cumming School of Medicine University of Calgary Calgary, AB Canada; 5 Addiction and Mental Health Alberta Health Services Edmonton, AB Canada; 6 Department of Computer Science Faculty of Science University of Alberta Edmonton, AB Canada; 7 Department of Occupational Therapy Faculty of Rehabilitation University of Alberta Edmonton, AB Canada; 8 Department of Child and Youth Care Faculty of Health and Community Studies MacEwan University Edmonton, AB Canada; 9 Faculty of Nursing University of Alberta Edmonton, AB Canada; 10 Department of Psychiatry Faculty of Medicine Dalhousie University Halifax, NS Canada

**Keywords:** posttraumatic stress injury, first responders, messaging, mobile phone, text-based intervention, Text4PTSI, Text4Wellbeing

## Abstract

**Background:**

First responders are confronted with traumatic events in their work that has a substantial toll on their psychological health and may contribute to or result in posttraumatic stress injuries (PTSIs) for many responders. Persons with a PTSI usually seek management therapies. Evidence indicates that digital delivery of these therapies is an innovative, efficient, and effective way to improve PTSI symptoms as an adjunct to in-person delivery.

**Objective:**

This project aims to implement and provide accessible, convenient, and economical SMS text messaging services, known as Text4PTSI and Text4Wellbeing, to first responders in Alberta, Canada; to prevent and improve the symptoms of PTSI among first responders; and to improve their overall quality of life. We will evaluate posttraumatic symptoms and the impact of Text4PTSI and Text4Wellbeing on stress, anxiety, and depression in relation to the correspondents’ demographic backgrounds.

**Methods:**

First responders who subscribe to Text4PTSI or Text4Wellbeing receive daily supportive and psychoeducational SMS text messages for 6 months. The SMS text messages are preprogrammed into an online software program that delivers messages to subscribers. Baseline and follow-up data are collected through online questionnaires using validated scales at enrollment, 6 weeks, 12 weeks, and 24 weeks (end point). In-depth interviews will be conducted to assess satisfaction with the text-based intervention.

**Results:**

We hypothesize that participants who enroll in this program will have improved PTSI symptoms; increased or improved quality of life; and significant reduction in associated stress, depression, and anxiety symptoms, among other psychological concerns. Improvement will be determined in comparison to established baseline parameters.

**Conclusions:**

This research will be beneficial for practitioners and will inform policy-making and decision-making regarding psychological interventions for PTSI. Lessons from this study will inform the scale-up of the intervention, a cost-effective, zero contact therapeutic option to manage PTSI.

**International Registered Report Identifier (IRRID):**

PRR1-10.2196/30680

## Introduction

### Background

First responders are personnel with specialized training to render care to patients who experience traumatic events (eg, acute illness, accidents, natural disasters, and terrorism) as the first point of contact. These personnel typically include firefighters, police officers, paramedics, corrections officers, and emergency health care workers, who often attend to persons in critical situations or conditions [1-3].

Posttraumatic stress injury (PTSI) is a mental health condition that first responders may experience at some point in their careers [2]. Some potentially traumatic events include motor vehicle accidents, fires, sexual assault, floods, and situations involving unexpected death, among others. These events can bring about injury and stress-related traumas that influence emotional well-being and prosperity [4]. Most first responders report experiencing multiple traumatic events in their lives [2,4,5]. Approximately 6.8% of people develop PTSI over their lifetime [6]. PTSI symptoms may affect the general well-being of the individuals; alter mood; and contribute to insomnia, irritability, and traumatic flashbacks [2].

PTSI is closely related to posttraumatic stress disorder (PTSD). Both share similar symptoms and are sometimes used interchangeably across the literature. Differences are subject to debate among various schools of thought. Whereas PTSD refers to a psychiatric disorder, PTSI, as defined by the Global PTSI Foundation, refers to biological injury associated with physical changes in the nervous system [7]. Psychiatrists and military officers have suggested that relinquishing the word “disorder” in favor of “injury” will minimize the stigma that prevents troops from seeking treatment. Thus, changing the name from PTSD to PTSI would change people’s perception of the condition [8].

PTSI rates are high among first responders ranging from one-third to more than half of those exposed to potentially traumatic events [9]. Globally, it is estimated that 10% to 35% of first responders experience psychological conditions, including PTSI [3,10]. A meta-analysis examining mental disorders among ambulance personnel estimated prevalence rates of 11% for PTSD, 15% for depression, 15% for anxiety, and 27% for general psychological distress [11,12]. Another study revealed that 80% of rescue, firefighter, medical, and police personnel who cared for victims of an apartment building explosion reported at least a symptom of PTSD [13]. Surprisingly, the prevalence rate of PTSD in these groups were found to widely range from 0% to 46% [14,15]. First responders experiencing PTSI may decrease productivity, increase risk of suicide, and show poor social interaction [16].

The most common co-occurring diagnosis with PTSI is depression, with about 36% to 55% of patients with PTSI experiencing concurrent depression [4,17]. A study conducted among first responders showed an increased risk of PTSI of 25.6% and 16.7% at 7 and 13 months, respectively, with a depression rate of 16%, after exposure to a traumatic event [18]. A proportion of first responders who are exposed to traumatic events seek psychological support or treatment [6,19]. There is no doubt that PTSI is a substantial mental health concern, especially among first responders, and contributes to a substantial overall cost for mental health. It is estimated that there are 69,000 police personnel; 110,000 firefighters; 30,000 paramedics; 17,000 correctional services personnel; over 7000 border services personnel; and 18,000 volunteer search and rescue personnel who serve as first responders in Canada [18]. About 2.5 million Canadian adults and 70,000 first responders have experienced PTSI in their lifetimes [9].

Psychological treatment to address PTSI may be unavailable due to access considerations such as location or lengthy waitlist, among others [1]. This informs the need for a service that will improve access and prevent and manage PTSI digitally. Narrative synthesis indicates that the digital delivery of these therapies might be as powerful as in-person delivery [20,21]. Additionally, these means may also lessen stigma and price while providing a remedy [22,23]. Evidence has shown that about one-third of persons with PTSI never recover due to inadequate and high-cost care; hence, cost-effective management is required [22]. Because of PTSI and its impact on first responders and their families, governments and other policy-making bodies, including the Canadian Workers’ Compensation Boards, initiated a guide for the rehabilitation of PTSI with a structured legislature to accept PTSI claims [23-25].

Mobile/smartphone technology can support and improve patient outcomes in psychological care [26,27]. An acceptable way to provide psychological interventions to the public and to those with mental health conditions is supportive SMS text messaging. Considering it is comparatively affordable, available, and efficient, text-based messaging can be delivered to many users at the same time, which may reduce the mental health treatment gap [26]. Mobile/smartphone messaging can prevent and manage health conditions and provide individuals with supportive and customized messages pertaining to their unique health conditions [26,28,29]. Supportive SMS text messaging can reduce hospitalizations and improve health behavior [30,31]. Research has demonstrated that, overall, an individual’s mental health improves upon receiving supportive SMS text messages [32-34]. Early provision of psychoeducation is effective in the prevention and management of PTSI [35]. About 80% of subscribers reported high satisfaction with the service and improved mental health status in studies conducted in Alberta [36]. A global survey conducted in 2015 revealed the median of access to the internet, with 87% having access to the internet and 68% of people own a smartphone. Among western countries, Canada is in a high-use group, with 90% of Canadians using the internet and 67% owning a smartphone [37]. These messages will effectively support patients who are attending individual or group treatment at Alberta Mental Health Centres.

Adjunct supportive text message therapy for psychological conditions has been tested on mood disorders among other psychological conditions [26]. However, to our knowledge, this innovation has not yet been conducted with PTSI and with first responders specifically. To address this gap, we propose an innovative program for first responders that seek to prevent and manage PTSI among this cohort with an evidence-based supportive SMS text messaging program developed using the concept of cognitive behavioral therapy.

### Objectives

This project will implement a structured daily SMS text messaging service known as Text4PTSI and Text4Wellbeing, which aims to prevent and reduce the occurrence of PTSI and to manage PTSI symptoms among first responders. We will evaluate posttraumatic symptoms; correspondent’s demography; and Text4PTSI’s and Text4Wellbeing’s impact on stress, anxiety, and depression.

We propose the following research questions:

- Will Text4PTSI and Text4Wellbeing prevent/improve the symptoms of PTSI among Alberta’s First Responders (eg, firefighters, police officers, paramedics, sheriffs, corrections officers, and emergency health care workers)?
- Will Text4PTSI and Text4Wellbeing improve the quality of life in the community of first responders experiencing PTSI symptoms by reducing symptom burden?
- How satisfied are subscribers with Text4PTSI and Text4Wellbeing programs (technical care, access, and utility)?

## Methods

### Study Design

The mixed methods study design will engage both quantitative and qualitative approaches [38]. Both approaches will be used independently as well as together and triangulated using the theoretical framework.

### Theoretical Framework

Outcomes have been defined as the change in health status directly attributable to the efforts or success of the health care experience [39-41]. Thus, successful treatment is associated with the efficacy of the provided care [42-45]. However, this study, as with many mental health impact evaluations, is subjective [46], given that it applies self-reported assessments by the participants. However, as suggested by the desire-fulfillment theory, outcomes possess a predominately positive association with satisfaction [47,48]. This is because the underlying rationale for seeking care is often to alleviate some form of functional impairment [49]. Comparing evidence on the difference (and similarities) between the need for health as perceived by the patient and the need for health care (as defined by the physician through scientific means) provides an opportunity for research. Consequently, this study will contribute to closing this gap by triangulating evidence from standardized psychological evaluation with evidence of patient satisfaction. Batbaatar and colleagues [50] identified nine determinants of variations in satisfaction with health care services: technical care, interpersonal care, physical environment, access (accessibility, availability, and finances), organizational characteristics, continuity of care, and outcome of care (Table 1). Our study will be guided by this framework and adapted for our e–mental health intervention (Table 2).

**Table 1:** Theoretical framework guided by Batbaatar et al’s [50] determinants of patients’ satisfaction.

Determinants	Batbaatar et al’s [50] definition	Text4PTSI/Text4Wellbeing adaptation
Technical care	The extent to which the services adhere to standards and norms of clinical diagnoses and treatments	Perception of the extent to which the services adhere to standards and norms of clinical diagnoses and treatments
Interpersonal care	The amount of caring for patients through noticing, participating, sharing, active listening, companioning, complimenting, comforting, hoping, forgiving, and accepting.	Not directly applicable
Physical environment	Pleasantness of the atmosphere, room comfort, bedding, cleanliness, noise level, temperature convenience, lighting convenience, food service, bathroom comfort, clarity of sign and directions, arrangement of equipment and facilities, and parking.	Not directly applicable
Access	Health service access is a multidimensional determinant measured by how (1) organizational issues (accessibility), (2) service resources (availability), and (3) personal barriers (affordability) prevent populations from access to health services.	(1) Ease of use of technology (text messaging services) and (2) personal barriers
Organizational characteristics	Reputation and image of the hospitals	Not directly applicable
Continuity of care	Uninterruptedness of health service process from the same hospital, location, or provider and in which the patient and the physician are cooperatively involved in ongoing health care management toward the goal of high quality, cost-effective medical care	(1) Continued use of intervention throughout the study period and (2) perceived complementary nature or linkage of Text4PTSI and Text4Wellbeing with participants existing mental health care
Outcome of care	Patients’ perceived health improvement	Patients’ perceived mental health improvement

**Table 2:** Gantt chart for Text4PTSI and Text4Wellbeing text message project.

Milestone accomplishment	Timelines (months)						
	1-2	3-4	5-6	7-8	9-10	11-12	13-15
Ethics approvals: an amendment of existing ethics approval for the Text4Support and Text4Hope programs to cover the Text4PTSI and Text4Wellbeing programs	✓^a^						
Preimplementation stakeholder engagement: stakeholder participation in content development and program advertisement	✓						
Technology/content development: Text4PTSI and Text4Wellbeing technologies and content development	✓						
Launch of Text4PTSI and Text4Wellbeing programs: Text4PTSI and Text4Wellbeing in operational use for first respondents to self-subscribe	✓	✓	✓	✓	✓		
Program evaluation: conducting quantitative and qualitative evaluation		✓	✓	✓	✓	✓	
Preliminary report: availability of preliminary program evaluation report				✓			
Postimplementation knowledge transfer activities: stakeholder engagement on preliminary findings and dissemination of early results						✓	
Final report development: developing final report and disseminated						✓	✓

^a^The checkmark indicates that this milestone will be accomplished at this time point.

### Inclusion Criteria

First responders who are 18 years or older who can provide informed consent will be eligible for the study. The Text4PTSI and Text4Wellbeing programs will be promoted to all first responders, including those who are healthy and may be seeking prophylactic psychological support to avert the onset of PTSI symptoms, those who might be experiencing PTSI symptoms but have not sought face-to-face psychological care, and those who are already accessing psychological care. First responders will also need to possess a mobile phone with an active line and have access to SMS text messages.

### Exclusion Criteria

First responders will be ineligible if they do not meet the aforementioned inclusion criteria or reside outside of regular cell phone connection areas.

### Recruitment

The first responders in Alberta (eg, firefighters, police officers, paramedics, corrections officers, and emergency health care workers) who regularly encounter difficult, risky, and traumatic events, and are willing to consent to the program will be targeted for recruitment. We will work with all first responder organizations to reach out to all first responders with information about the program. In addition, Text4PTSI and Text4Wellbeing would be the subject of a wide-exposure communications campaign (TV, radio, internet, and print media). First responders at risk of PTSI and those experiencing PTSI symptoms and on a waitlist to obtain care through the Alberta Health Services centralized Addiction and Mental Health intake service and the Operational Stress Injury Clinic with extra passageways such as private psychologists and counsellors will be included as the project evolves.

### The Text4PTSI and Text4Wellbeing Interventions

First responders will participate in the Text4PTSI and Text4Wellbeing programs by simply texting “PTSI” and “Wellbeing,” respectively, to a designated phone number. Text4PTSI and Text4Wellbeing will offer quick support to first responders encountering PTSI symptoms, including individuals who are on a waitlist for services or may experience issues getting to support because of geographic boundaries or other access considerations. Beginning on the first day of enrollment, first responders will receive daily unidirectional computer-programmed supportive SMS text messages, which will be specifically designed to alleviate PTSI symptoms. The messages will be written by cognitive behavior therapists and psychologists who have experience in treating PTSI in partnership with a cross section of first responders receiving treatment at the Operational Stress Injury and Addiction and Mental Health clinics in Edmonton and Calgary. The messages will be preprogrammed into a software program that will deliver the messages to subscribers automatically. The content of the SMS text message will focus on two dimensions. The first dimension includes general content that is indicated regardless of symptom presented, including messages of self-care, social support, hope, affirmation, and recovery. In the second dimension, specific content will be provided that focuses on the management of PTSI-related symptoms and conditions, including PTSD, generalized anxiety disorder, panic disorder, major depressive disorder, and suicidal ideation (eg, activity scheduling in depression). The ultimate goal will be to have SMS text message content customized based on end user characteristics. To this end, over time, we will appraise the individual needs of participants based on occupational category, age group, cultural identity, sex, gender identity and gender expression, sexual orientation, ethnicity, culture, religious perspective, income, and non-English language communication. This will be in accordance with the user identified needs and may enhance access for numerous groups, many of whom may be marginalized or underserved. For the program under study, the SMS text messages will target specific PTSI risk factors and symptoms with consideration for occupational and sociocultural context. An example [51] is:

Letting go of resentment is a gift you give yourself, and it will ease your journey immeasurably. Make peace with everyone, and happiness will be yours. Trauma can feel like a gloomy cloud over all areas of your life. The first step in treatment is to understand what trauma is, the symptoms, and how and why it is treated.

### Sample Size Considerations

With Alberta’s first responder population of about 33,000 [52], a 95% CI, and a 3% margin of error, the sample size needed for prevalence estimates for likely PTSI will be 1034. Based on baseline survey response rates of 20% achieved with both Text4Mood and Text4Hope programs [26,53,54], to achieve our sample size target, we plan to enroll 5170 first responders in the Text4PTSI and Text4Wellbeing programs.

### Data Collection

At the beginning of the first message, respondents will be introduced to the program by completing an online mental health assessment, which will be repeated at 6 weeks, 3 months, and upon completion of the program at 6 months. A similar approach was successfully used to collect cross-sectional and longitudinal data from thousands of Text4Hope subscribers during the COVID-19 pandemic [53-65]. In-depth interviews on participant satisfaction with Text4PTSI and Text4Wellbeing will be conducted involving a cross-section of 10 to 15 participants selected randomly using an interview guide informed by the theoretical framework.

### Outcome Measures

Key outcomes of interest in this study include change in scores measured by comparing pre- vs posttreatment responses to the following surveys: PTSD Checklist 5 [66], Generalized Anxiety Disorder-7 scale [67], Perceived Stress Scale [68], and the Patient Health Questionnaire-9 [69]. Secondary outcomes will include quality of life, as measured with the Well-being Index [70], and program satisfaction measured with the Text4PTSI and Text4Wellbeing exit questionnaire.

A patient satisfaction questionnaire will be programmed to measure the applicability of the technology as a valuable modality for delivering psychological therapies to large numbers of first responders experiencing PTSI symptoms. It is also designed to assess the benefits of the program to service users. A patient satisfaction questionnaire previously used to evaluate the Text4Mood program and Text4Hope program [26,55] will be adapted to evaluate the Text4PTSI and Text4Wellbeing Programs. As part of the sociodemographic variables, the satisfaction questionnaire will address multiple measures of satisfaction, including subscriber-reported effects of the program on symptoms and quality of life, and the usability of the technology. Table 3 illustrates an overview of the Text4PTSI and Text4Wellbeing outcome measures.

**Table 3:** Overview of the Text4PTSI and Text4Wellbeing outcome measures.

Outcome		Instrument		Time		Data source
**Effectiveness**						
	Quality of life	5-Item World Health Organization Well-being Index	These measures will be assessed at baseline, 6 weeks, 3 months, 6 months, and 12 months		Clinical questionnaire	
	Depression symptom score	Patient Health Questionnaire-9	These measures will be assessed at baseline, 6 weeks, 3 months, 6 months, and 12 months		Clinical questionnaire	
	Anxiety symptom scores	Generalized Anxiety Disorder-7	These measures will be assessed at baseline, 6 weeks, 3 months, 6 months, and 12 months		Clinical questionnaire	
	Participants perceived stress	Perceived Stress scale	These measures will be assessed at baseline, 6 weeks, 3 months, 6 months, and 12 months		Clinical questionnaire	
	PTSD^a^ symptom score	PTSD Checklist	Assessed at baseline, 6 weeks, 3 months, 6 months, and 12 months		Clinical questionnaire	
	Client satisfaction/experience surveys	Instrument developed and pilot tested, and published by the authors	Assessed at 6 weeks, 3 months, 6 months, and 12 months		Survey questionnaire	
**Implementation**						
	Reach	The proportion of first responders who receive the daily supportive text message	Assessed at baseline, 6 weeks, 3 months, 6 months, and 12 months		Survey questionnaire	
	Acceptability	Instrument developed and pilot tested, and published by the authors	Assessed at 6 weeks, 3 months, 6 months, and 12 months		Survey questionnaire	
	Fidelity	Part of first responders who read the supportive text messages at least once a day/percentage of scheduled follow up	Assessed at 6 weeks, 3 months, 6 months, and 12 months		Survey questionnaire	
	Cost	Administrative data	Assessed at baseline and 12 months		N/A^b^	

^a^PTSD: posttraumatic stress disorder.

^b^N/A: not applicable.

### Data Analysis

A quantitative evaluation will be used with a descriptive and inferential analytical approach. The analysis will demonstrate the distribution of likely PTSI with respect to sociodemographic characteristics of participants, as well as identify determinants of likely PTSI symptoms and outcomes across various predictor variables. Using subscriber data from the overall study, an additional benefit will be the application of machine learning to develop an artificial intelligence approach to predict characteristics and risk factors of first responders who commonly experience PTSI symptoms. This would allow us to generate predictive tools to differentially support first responders at risk of PTSI. This activity will be achieved through collaboration with the computational psychiatry group. This approach is well within the expertise of the computational psychiatry group members and in line with PTSI/PTSD trauma-related care [71,72].

Qualitative data will be analyzed to explore respondents’ preferences, satisfaction, and other characteristics that will help improve outcome, program design, preventive strategies, and workplace and government policies as they relate to PTSI. Qualitative data analysis will involve inductive and deductive thematic analytic approaches using the study’s theoretical framework. Qualitative descriptive methodology is an appropriate approach for qualitative research geared toward generating information to refine interventions in everyday terms [73]. In accordance with qualitative descriptive methods [73], qualitative content analysis will be conducted to summarize the content of the data [74].

What is also unique about this study is the demographic data collected from users. Collecting this information will also allow us to engage in comparative analysis, which may reveal new patterns or trends in the mental health of first responders based upon how they identify. This will allow the research team to further customize text-based supportive messages based on characteristics including age, gender, race, sexual orientation, etc.

### Ethical Considerations

The study received ethics approval from the Health Research Ethics Board (HREB) of the University of Alberta (Pro00108966). Informed consent will be obtained from all participants. Confidentiality and data security measures will be adhered to as approved by the HREB.

## Results

We hypothesize that participants who will enroll in this program will have improved quality of life; reduced PTSI symptoms; and significantly improved associated moderate/high stress, depression, and anxiety symptoms, among others. Improvement will be in comparison to the baseline parameters.

## Discussion

The nature of first responders’ work impacts their health, daily activities, and psychological safety and well-being. The psychological impact on this cohort requires innovative, technologically driven psychological supports that have no waitlists, are geographic location independent, and can serve first responders at risk and those experiencing PTSI symptoms, while respecting confidentiality and reducing stigma. This approach will mitigate the potential adverse effects of first responders accessing psychological care. If Text4PTSI and Text4Wellbeing are effective for first responders in Alberta, we will explore opportunities for a national scale-up and global dissemination through engagements with first responder organizations as well as regional and national governments. This would be achieved through a Canadian federally registered not-for-profit organization, the Global Psychological eHealth Foundation [75] working in partnership with the research team. A similar program (Text4Mood) implemented in Alberta demonstrated an improved psychological treatment gap [26]: 77% of the participants felt capable of managing depression and anxiety, while 83% had improved overall mental well-being. Likewise, the Text4Hope program launched in Alberta during the COVID-19 pandemic reduced mental health distress by about 20% in subscribers [51]. It is expected that Text4PTSI and Text4Wellbeing supportive SMS text messages that seek to address access gaps to psychological care for first responders in Alberta and those experiencing or at risk of experiencing PTSI symptoms will achieve results comparable to the Text4Mood and Text4Hope programs.

Text4PTSI and Text4Wellbeing supportive SMS text messages seek to address access gaps to psychological care for first responders in Alberta and those experiencing PTSI symptoms. The proposed mobile technology intervention is a potentially effective, economical, and accessible way of providing an intervention to first responders. Previous studies have proven positive outcomes with high satisfaction with the delivery of supportive messages [26]. Thus, Text4PTSI and Text4Wellbeing could alleviate onerous challenges of access to care for first responders, considering individual location, unique demographic considerations, financial constraints, stigma, and position on the waitlist for those already being managed for PTSI [76]. This project will provide support to the first responders irrespective of geographical location or identity.

This project will assess outcomes with standard validated questionnaires and provide essential statistics regarding the prevalence of PTSI among first responders in Alberta. Thus, information from this project will be beneficial for practitioners and important for policy- and decision-making regarding psychological interventions for PTSI in the target population.

The project will promote partnerships and networks among mental health stakeholders to improve knowledge and understanding of the social issues and challenges faced by first responders. The research and evaluation aspect of the project may scale up effective approaches to identify and respond to existing and emerging social issues that first responders and their families confront.

A potential limitation of the study design is that there may be selection bias since phone service coverage may be unstable in some rural remote locations in Alberta, which may skew the findings toward a more urban population. Another limitation is that the prevalence of anxiety, depression, and PTSI to be reported in this study is based on standardized self-rated scales rather than clinical interviews using the Diagnostic and Statistical Manual for Mental Disorders (Fifth Edition). These limitations notwithstanding, this study will provide valuable data about the prevalence of anxiety, depression, and PTSI symptoms in first responders and their correlates, as well as the impact of Text4PTSI and Text4Wellbeing on anxiety, depression, and PTSI symptoms in first responders. Similar to results achieved with earlier supportive SMS text messaging programs, we expect Text4PTSI and Text4Wellbeing programs to improve the mental well-being among first responders.

## Abbreviations

HREB: Health Research Ethics Board
PTSI: posttraumatic stress injury
PTSD: posttraumatic stress disorder
